# Supplementary material for: Circulating tumour DNA reflects treatment response and clonal evolution in chronic lymphocytic leukaemia
Source: Nat Commun. 2017 Mar 17;8:14756. doi: 10.1038/ncomms14756 (PMC5357854; doi:10.1038/ncomms14756)
Supplement: Supplementary Information — Supplementary Figures and Supplementary Tables [file ncomms14756-s1.pdf]

Supplementary Figure 1

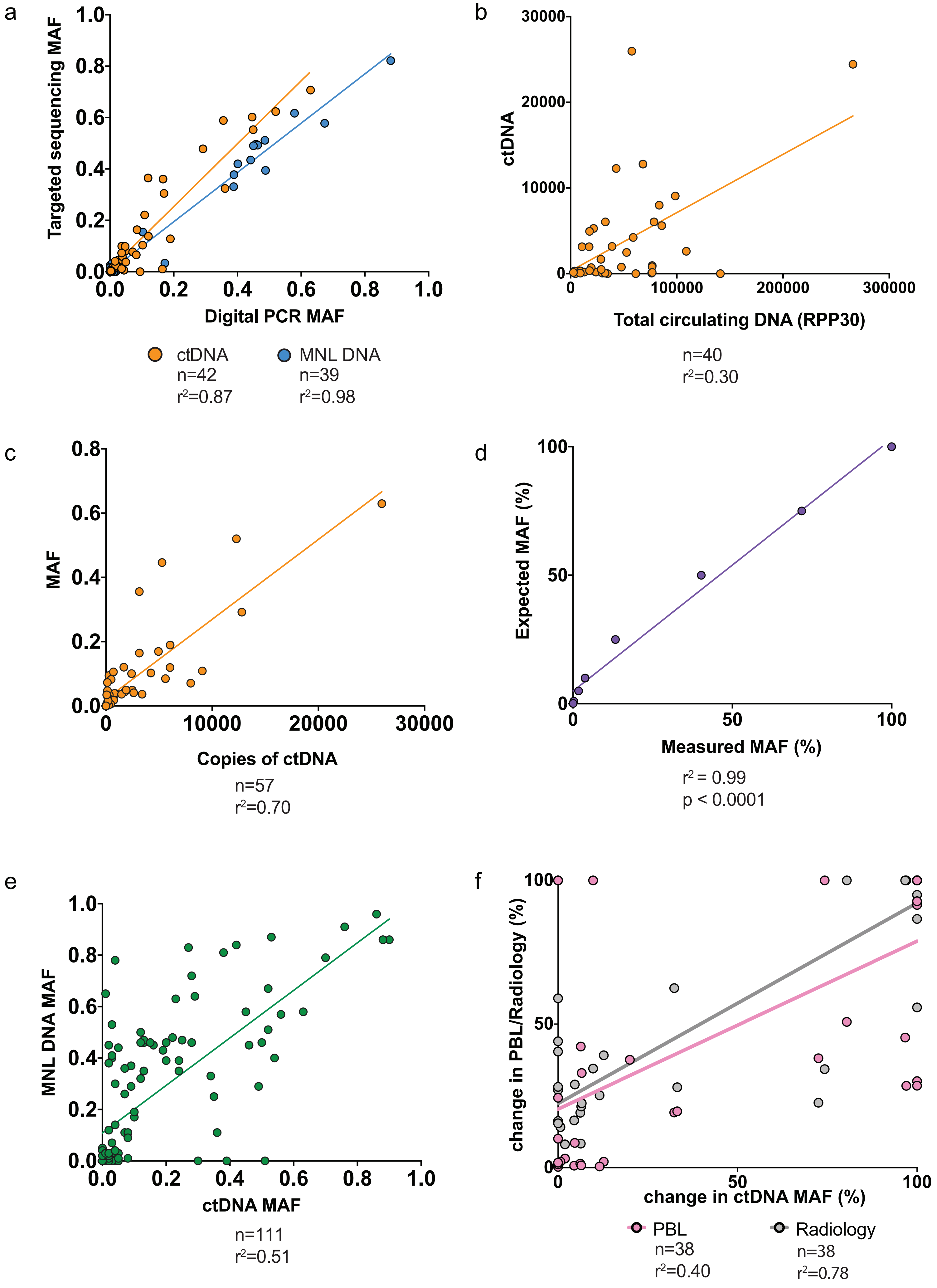

Supplementary Figure 1: Correlation of quantitative data from digital PCR and targeted sequencing (TS)

(a) Mutant allele fractions (MAF) of 11 mutations across all cases obtained separately using digital PCR and TS were compared. MAFs obtained using both techniques showed strong correlation for both ctDNA (r^2=0.87 p<0.0001) and MNL DNA (r^2=0.98 p<0.0001) respectively.

(b) Comparison of the absolute copies of total cell-free DNA (calculated based on copies of the *RPP30* gene), and the absolute copies of mutant ctDNA for 7 mutations across all cases obtained using digital PCR, showed minimal correlation (r^2=0.30, p=0.0003).

(c) Number of absolute copies of mutant ctDNA showed a strong correlation (r^2=0.70 p<0.0001) with their corresponding MAF for 11 mutations across all cases, as assessed by digital PCR.

(d) Serial dilution of OVCAR cell line DNA (harboring a *TP53* R248Q mutation) into wild-type DNA, such that the MAF ranged from 0.1% to 99%. Digital PCR was used to analyze the serial dilutions, showing strong correlation between the expected and measured MAF (r^2=0.99 p<0.0001) across this range.

(e) Correlation between MAFs observed in ctDNA and MNL DNA across 111 different time-points across the series (r^2=0.51 p<0.0001).

(f) Comparison of the change in peripheral blood lymphocyte (PBL) count or the change in radiological disease burden (cm^2) with the change in ctDNA MAF, from the maximal value analysed for each individual patient. Of 38 matched time-points, changes in ctDNA showed a stronger correlation with changes in radiological disease burden (r^2=0.78, p<0.0001) compared to changes in PBL count (r^2=0.40, p<0.0001).

All correlation analyses were performed using the Spearman method on GraphPad Prism 7.0.

Supplementary Figure 2

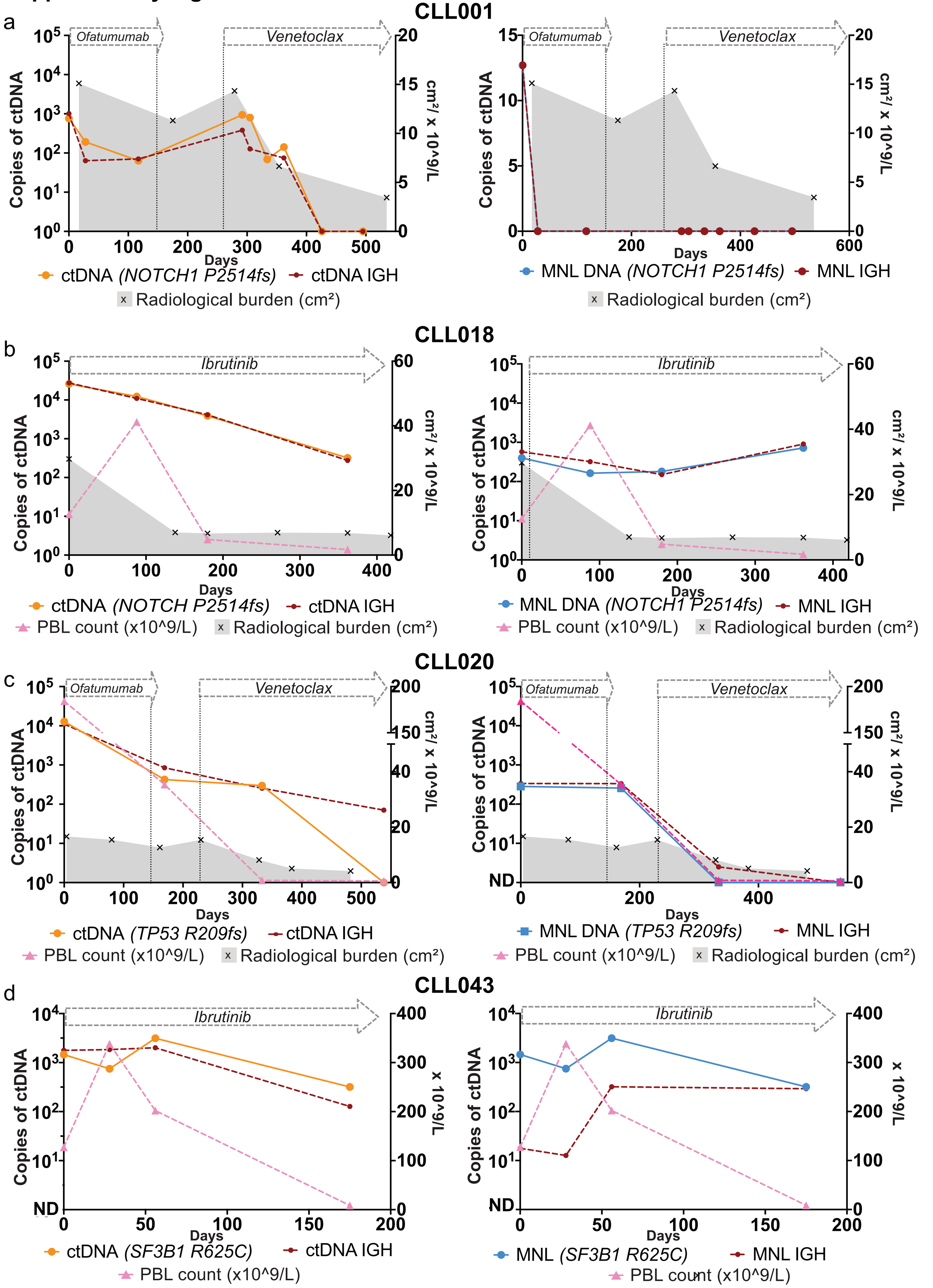

Supplementary Figure 2: ctDNA dynamics using patient-specific IGH rearrangements

Patient specific IGH rearrangements were analysed using digital PCR and the dynamics are shown for patients CLL001, CLL018, CLL020 and CLL043 (a-d). In all 4 patients, the serial changes in IGH levels in both ctDNA (left) and MNL DNA (right) tracked similarly with respective ctDNA and MNL DNA somatic mutation levels.

Supplementary Figure 3

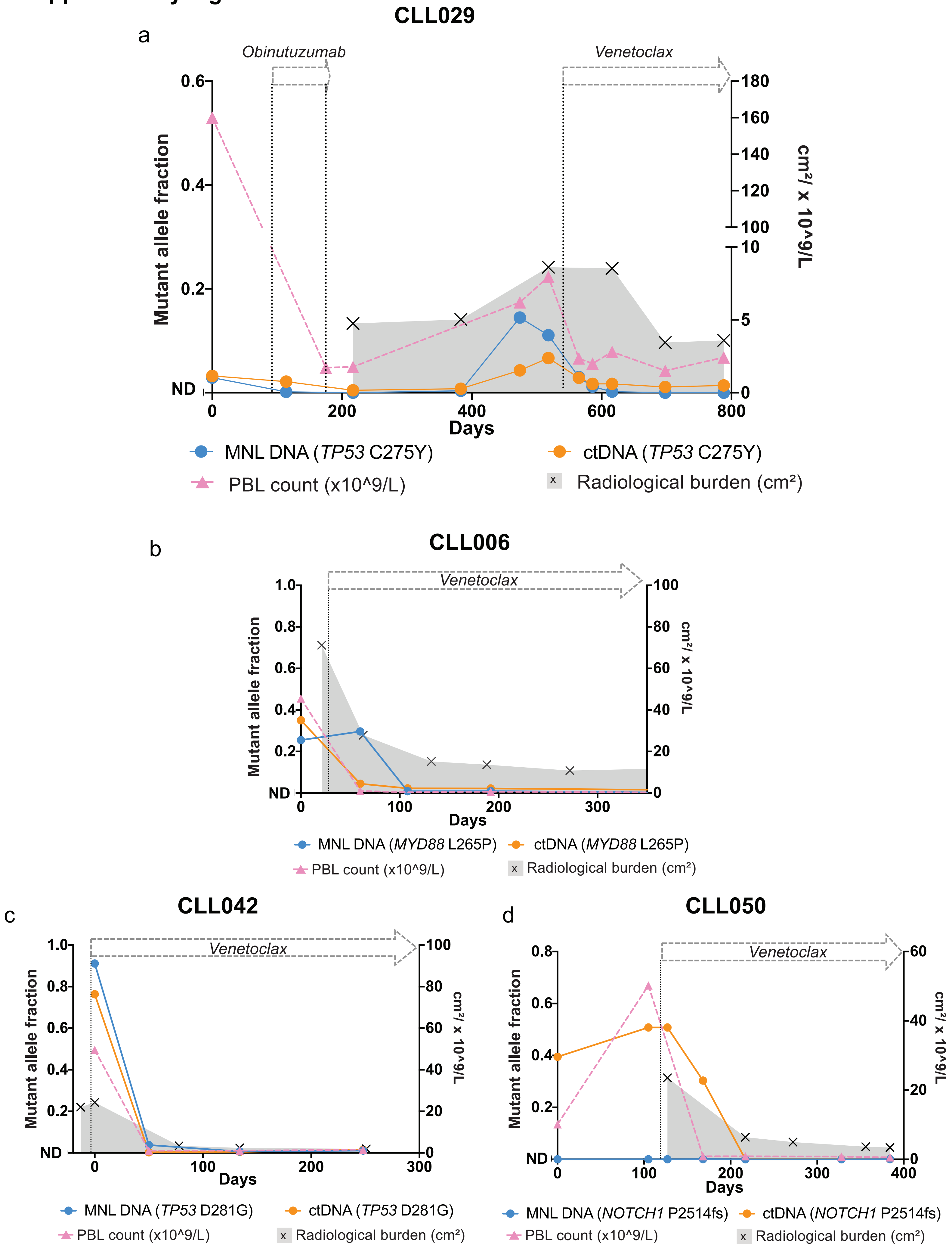

Supplementary Figure 3: ctDNA dynamics and clinical assessment in refractory CLL patients treated with venetoclax

(a) Case CLL029 had marked lymphocytosis and neck lymphadenopathy, which after 4 cycles of obinutuzumab maintained a partial response for 11 months before the patient progressed. Venetoclax subsequently induced a response with only MRD detected by flow cytometry. ctDNA MAF of the *TP53* mutation reflected the changes in disease state throughout Obinutuzumab and venetoclax therapy.

(b) Case CLL006 had widespread disease in lymph nodes, marrow and a moderate lymphocytosis. Following venetoclax these changes largely resolved with only MRD detected by flow in the bone marrow. ctDNA MAF of the *MYD88* mutation reflected the clinical response to venetoclax.

(c) Case CLL042 had extensive nodal, marrow and circulating disease, which responded well to venetoclax. ctDNA MAF of the *TP53* mutation reflected the clinical response to venetoclax.

(d) Case CLL050 had lymphocytosis and extensive marrow burden, which responded to venetoclax, resulting in complete remission and no MRD detected by flow cytometry. ctDNA MAF of the *NOTCH1* mutation reflected the clinical response to venetoclax. This was in contrast with the MNL DNA where the *NOTCH1* mutation was undetectable throughout.

Supplementary Figure 4

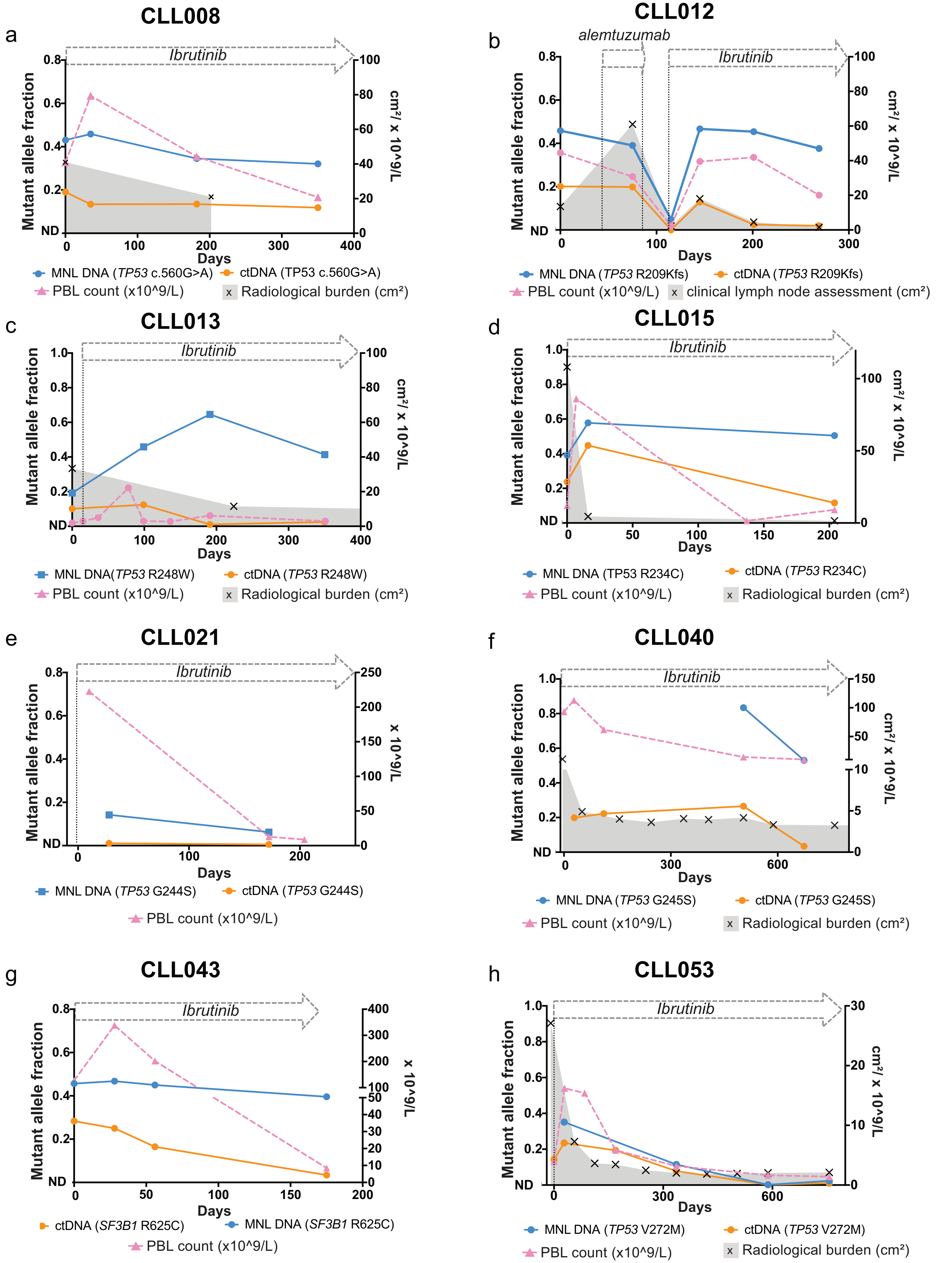

Supplementary Figure 4

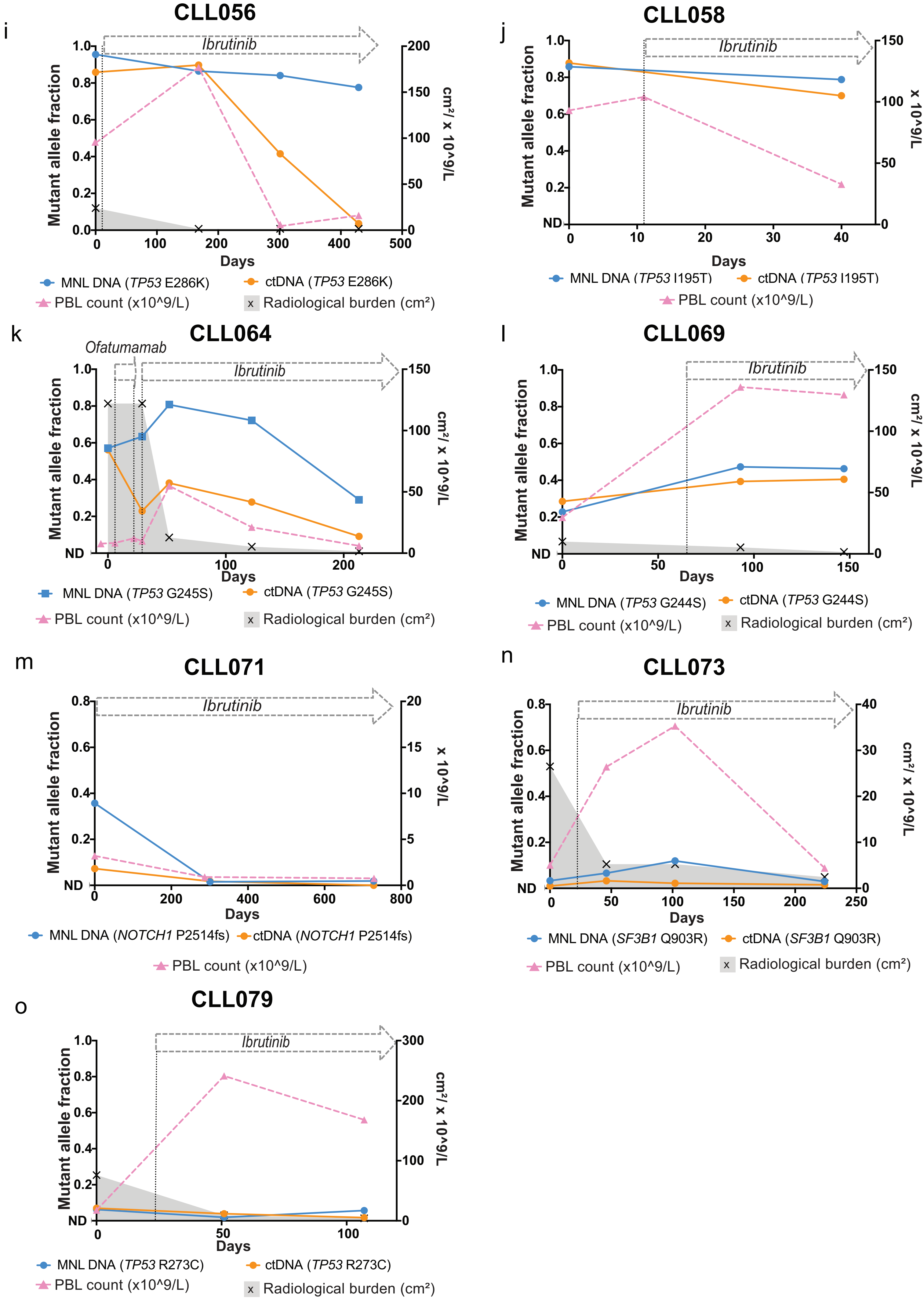

Supplementary Figure 4: ctDNA dynamics and clinical assessment in refractory CLL patients treated with ibrutinib

(a) Case CLL008. The reduction in lymphadenopathy was paralleled by decreasing levels of ctDNA (*TP53* mutation), despite an ibrutinib-induced spike in the PB lymphocyte count shortly after the commencement of treatment.

(b) Case CLL012. Patient was treated with alemtuzumab (campath) with a good initial response but developed complications and was switched to ibrutinib. Following ibrutinib, there was an overall partial response, although worsening lymphocytosis and a transient rise in lymphadenopathy (as assessed clinically by the treating hematologist) was present initially. These dynamics were represented by ctDNA MAF of the *TP53* mutation.

(c) Case CLL013. Patient had relapsed CLL in the abdomen and marrow. Ibrutinib therapy resulted in a complete remission after an initial worsening of the lymphocytosis. ctDNA MAF of the *TP53* mutation was initially stable before falling. This is in contrast to the MNL DNA MAF of *TP53* mutation, where the level showed an initial rise with the lymphocytosis before subsequently falling.

(d) Case CLL015. Patient had bulky lymphadenopathy at baseline. Ibrutinib therapy achieved a partial response after an initial worsening of the lymphocytosis. ctDNA MAF of the *TP53* mutation showed an initial rise, however subsequently fell with continued therapy to reflect the patient response.

(e) Case CLL021. Patient had lymph node and marrow disease with lymphocytosis, which responded rapidly after ibrutinib was introduced. ctDNA only analysed after the introduction of ibrutinib showed a *TP53* mutation at low levels (1% MAF).

(f) Case CLL040. Patient had widespread lymphadenopathy and lymphocytosis. Following the commencement of ibrutinib, there was radiological improvement in disease. An initial lymphocytosis was seen before a gradual reduction in lymphocyte count. ctDNA levels for the *TP53* mutation initially remained steady throughout therapy before eventually falling.

(g) Case CLL043. The kinetics of ctDNA as assessed by the decreasing MAF of the *SF3B1* mutation in plasma paralleled the concomitant decline in lymphadenopathy (as assessed clinically by the treating hematologist). This was despite a sharp increase in the PB lymphocyte count shortly after the commencement of ibrutinib treatment.

(h) Case CLL053. Patient had lymphadenopathy and extensive marrow burden, which responded well to ibrutinib after an initial worsening of lymphocytosis. Although the ctDNA level for the *TP53* mutation showed a small transient rise 28 days into ibrutinib therapy, there was reduction in the ctDNA at subsequent time-points reflecting the overall response to treatment.

(i) Case CLL056. Patient had extensive lymphadenopathy and marked lymphocytosis at baseline. Following ibrutinib therapy, there was a partial response with persisting mild lymphocytosis. ctDNA MAF for the *TP53* mutation showed stable levels despite the initial spike in PBL count and subsequently fell with continued therapy reflecting the treatment response.

(j) Case CLL058. Patient had extensive marrow involvement and lymphocytosis. Following ibrutinib, there was a modest reduction in PBL after an initial worsening of lymphocytosis. ctDNA levels for the *TP53* mutation showed an overall decrease in MAF after ibrutinib therapy.

(k) Case CLL064. Patient had moderate lymphocytosis and lymphadenopathy, which responded to ibrutinib after prior treatment with ofatumumab. Compared to baseline, there was an overall reduction of ctDNA levels of the *TP53* mutation consistent with treatment response.

(l) Case CLL069. Patient had marked lymphocytosis and mild lymphadenopathy. Following ibrutinib therapy there was an initial worsening of lymphocytosis but overall stable disease, which was reflected in the ctDNA MAF of the *TP53* mutation.

(m) Case CLL071. Patient had marked lymphadenopathy and moderate marrow burden at baseline. Following ibrutinib, there was reduction in lymphadenopathy by clinical examination with only MRD detected by flow cytometry. This was represented by a fall in ctDNA MAF of the *NOTCH1* mutation.

(n) Case CLL073. Patient had modest lymphadenopathy and heavy marrow burden at baseline. Following ibrutinib, there was an overall reduction in lymphadenopathy with an initial spike in lymphocyte count. ctDNA levels of the *SF3B1* mutation showed a reduction in MAF following the commencement of therapy.

(o) Case CLL079. Patient had bulky and widespread lymphadenopathy and mild lymphocytosis at baseline. Following ibrutinib, there was a PBL spike but a resolution in lymphadenopathy. ctDNA levels of the *TP53* mutation showed a gradual reduction in MAF following therapy.

Supplementary Figure 5

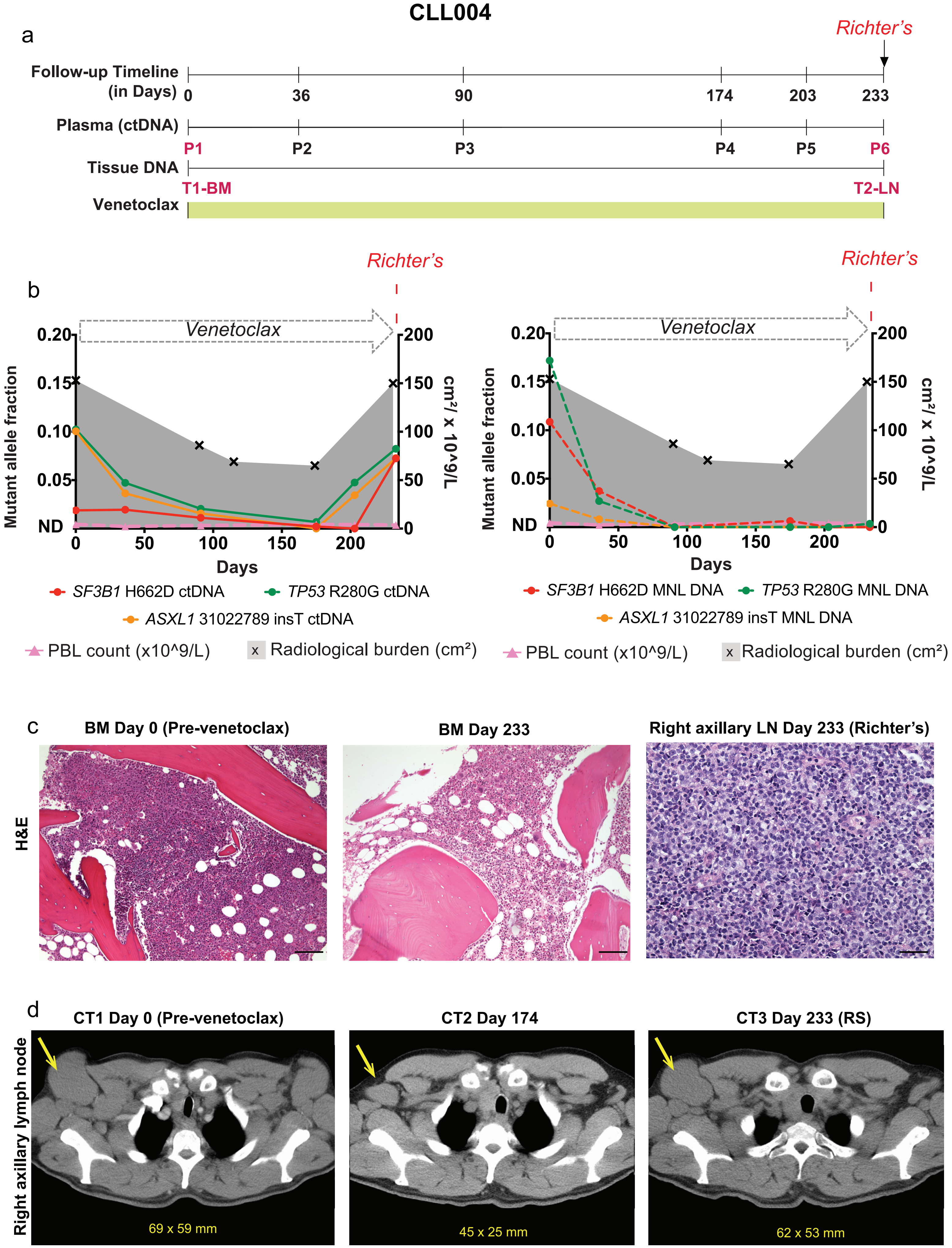

Supplementary Figure 5

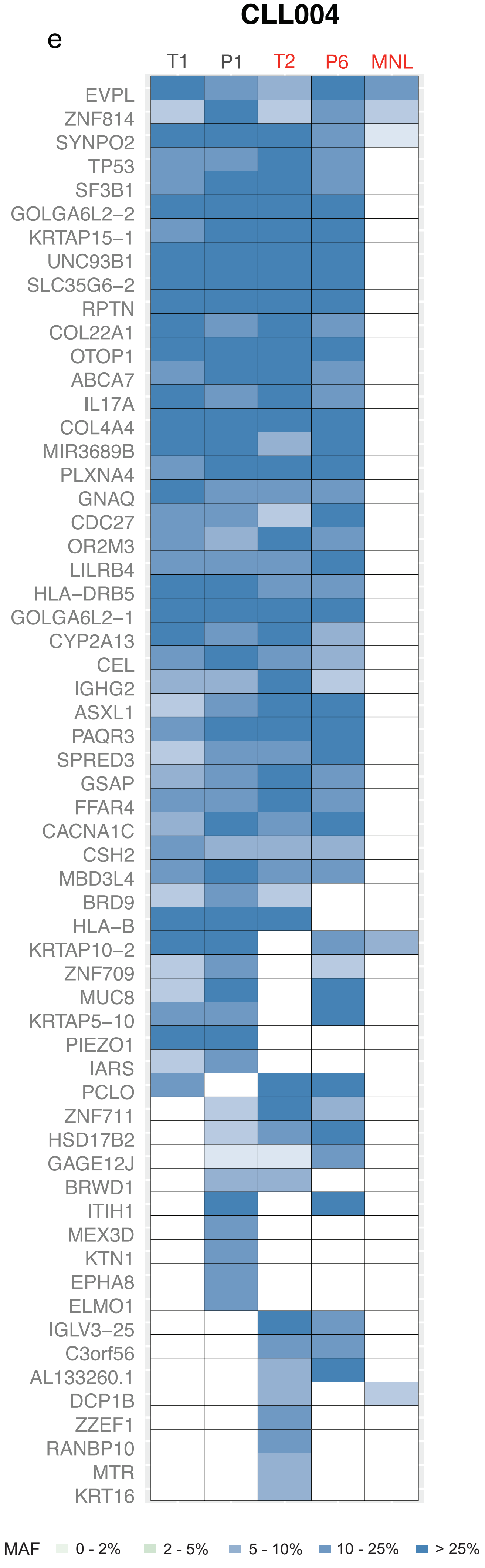

Supplementary Figure 5: Clinical assessment and ctDNA analysis in case CLL004

(a) A treatment timeline of case CLL004 highlighting the time-points of plasma (P) assessment and bone marrow (BM) / lymph node (LN) biopsies, respective to treatment with venetoclax. Whole exome sequencing (WES) was performed on DNA samples highlighted in red at baseline diagnosis (P1 and T1-BM) and at transformation to RS (P6 and T2-LN) in patient CLL004. Low-coverage whole genome sequencing (LC-WGS) was also performed on plasma (P1 and P6).

(b) ctDNA (Left) and matched MNL DNA (Right) dynamics were followed serially in case CLL004 and compared to clinical parameters of disease burden (PB lymphocyte counts and radiological measurement of lymphadenopathy). A rise in the fractional abundance of ctDNA, assessed by the dynamics of 2 mutations (*TP53* and *SF3B1*), was observed 30 days prior to the clinical diagnosis of RS in case CLL004. An increase in fractional abundance of an *ASXL1* mutation was seen at the time of diagnosis of RS. These *TP53, SF3B1*, and *ASXL1* mutations were not seen in matched MNL DNA at the time of RS.

(c) H&E staining of bone marrow biopsy (T1-BM) prior to the commencement of venetoclax displayed a high infiltration of CLL cells indicating heavy disease burden. In contrast, the bone marrow biopsy on Day 233 post-treatment (time-point of RS diagnosis) was normocellular with moderate CLL infiltration. H&E staining of the right axillary lymph node biopsy (T2-LN) displayed a dense distribution of larger B cells, indicating Richter's transformation at this site. Scale bar – 100μm.

(d) Representative CT images of the right axillary lymph node of case CLL004 prior to venetoclax treatment (Day 0), at Day 174 displaying reduced lymphadenopathy, and finally at Day 233 showing increased disease burden at the time of RS diagnosis.

(e) A heat-map illustrating the distribution of predicted functional SNVs from WES at baseline (BM and P) and at progression to RS (LN, P and matched MNL shown in red) in CLL004.

Supplementary Figure 6

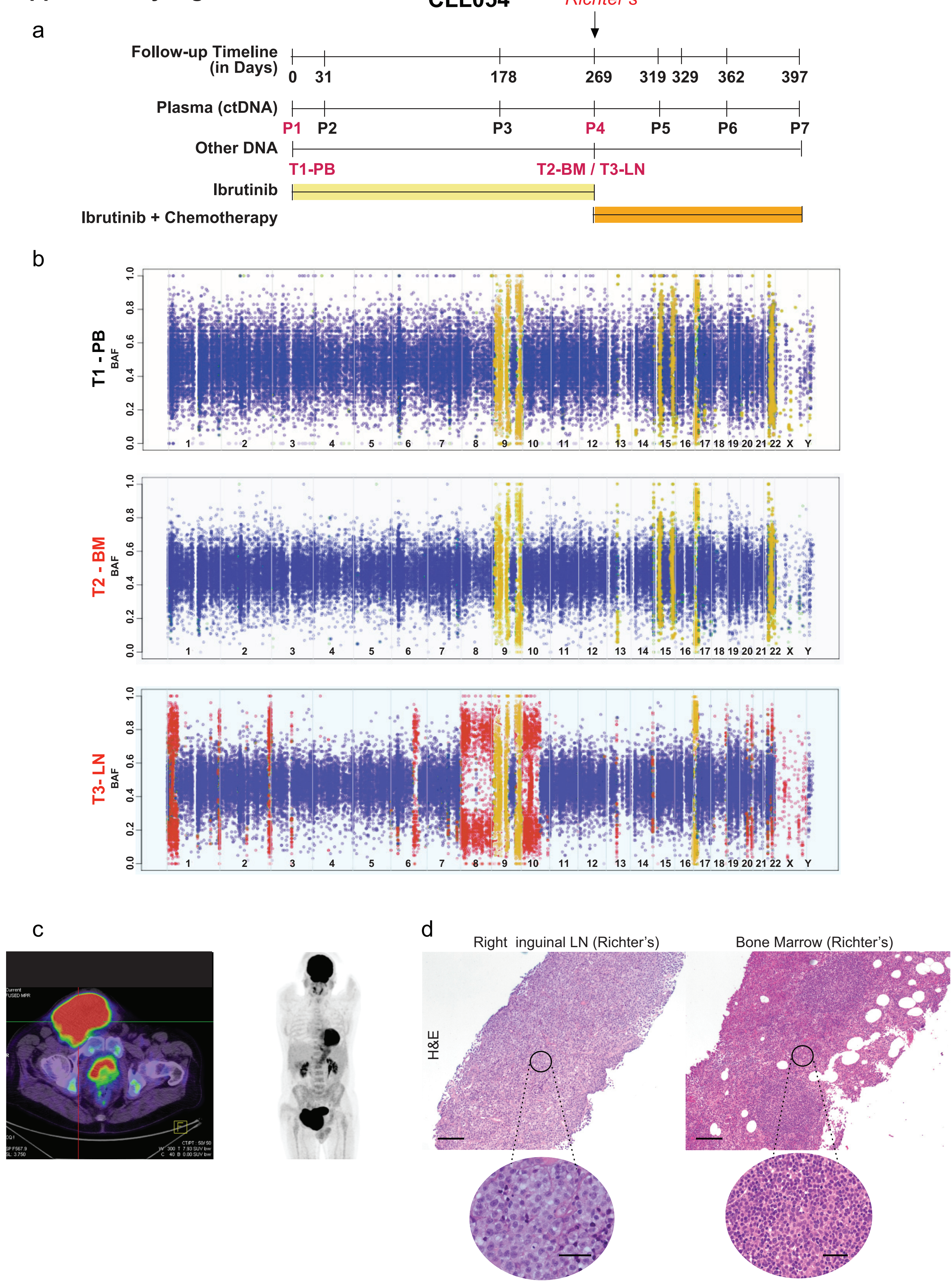

Supplementary Figure 6

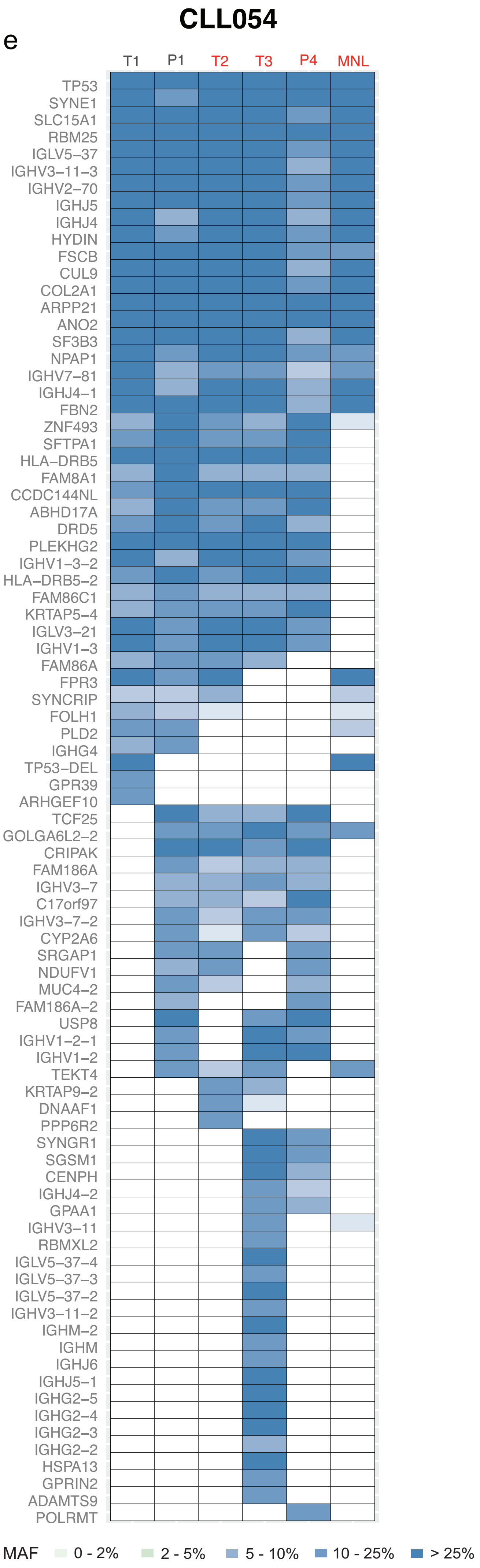

Supplementary Figure 6: Clinical assessment and ctDNA analysis in case CLL054

(a) A treatment timeline of case CLL054 highlighting the time-points of plasma (P) assessment and peripheral blood (PB) / bone marrow (BM) / lymph node (LN) biopsies, respective to treatment with ibrutinib and a combination of ibrutinib + chemotherapy. Whole exome sequencing (WES) was performed on DNA samples highlighted in red at baseline (P1 and T1-PB) and at the time of RS (P4, T2-BM and T3-LN). Low-coverage whole genome sequencing (LC-WGS) was also performed on plasma (P1 and P4).

(b) B-allele frequency (BAF) plots showing copy number alterations (CNAs) in WES from samples at baseline (T1-PB) and matched tissue samples at progression to RS (T2-BM and T3-LN). Marked in yellow are CNAs that were identified from WES of the CLL tissue samples (T1-PB and T2-BM), and red signifies new CNAs that were unique to T3-LN at the time of RS diagnosis.

(c) FDG-PET imaging at RS diagnosis (Day 269) showing a large right inguinal mass with intense uptake highly suspicious of transformed disease.

(d) H&E staining of right inguinal lymph node and bone marrow biopsy at time of Richter's transformation. The right inguinal node showed a confluent large cell infiltrate consistent with large cell lymphoma. The bone marrow biopsy showed a predominant infiltrate of CLL. Scale bar – 100μm (top panel) and 20μm (bottom panel).

(e) Heat-map illustrating the distribution of predicted functional SNVs from WES at baseline (PB and P) and at progression to RS (BM, LN, P and matched MNL shown in red) in CLL054.

Supplementary Figure 7

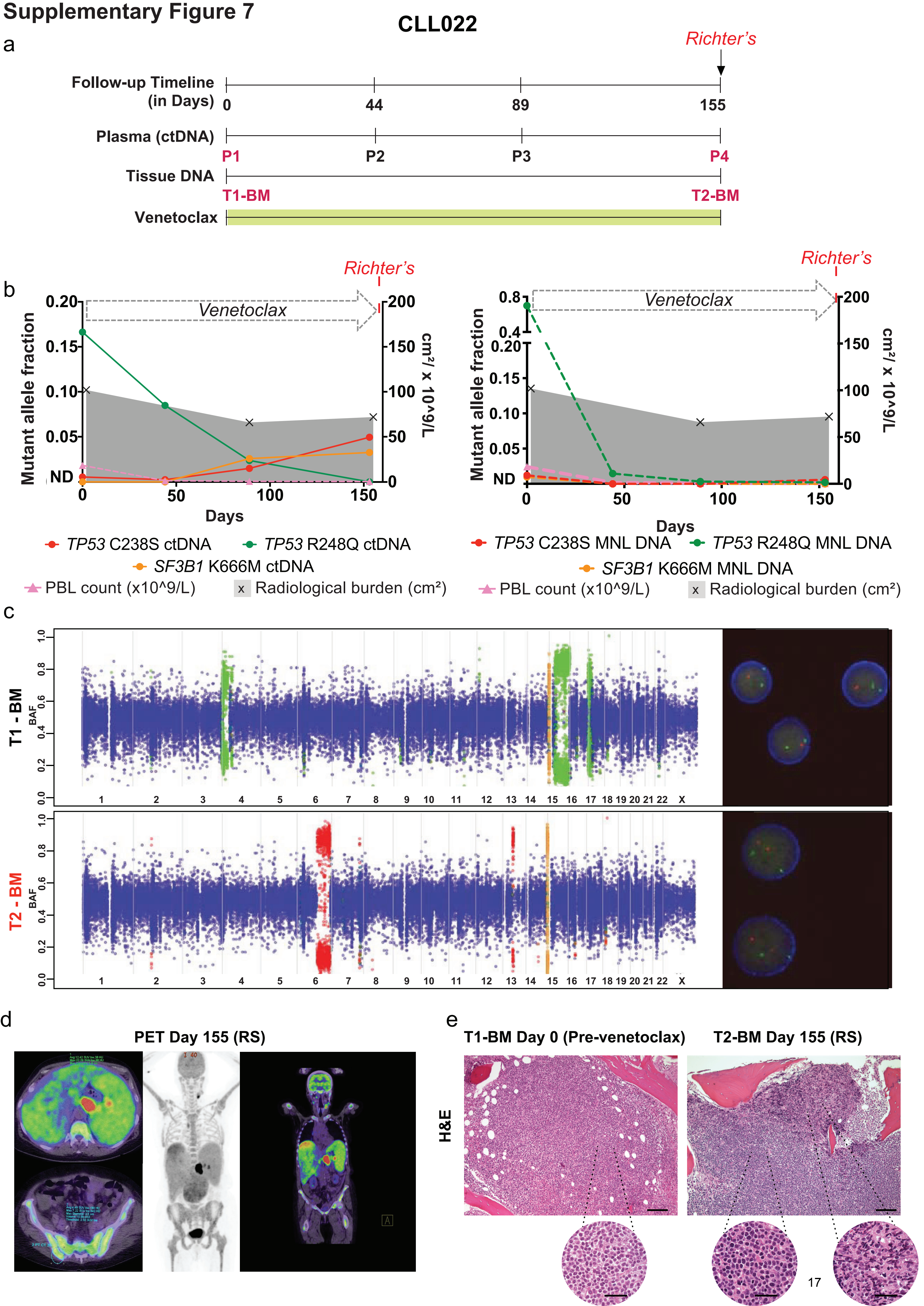

Supplementary Figure 7

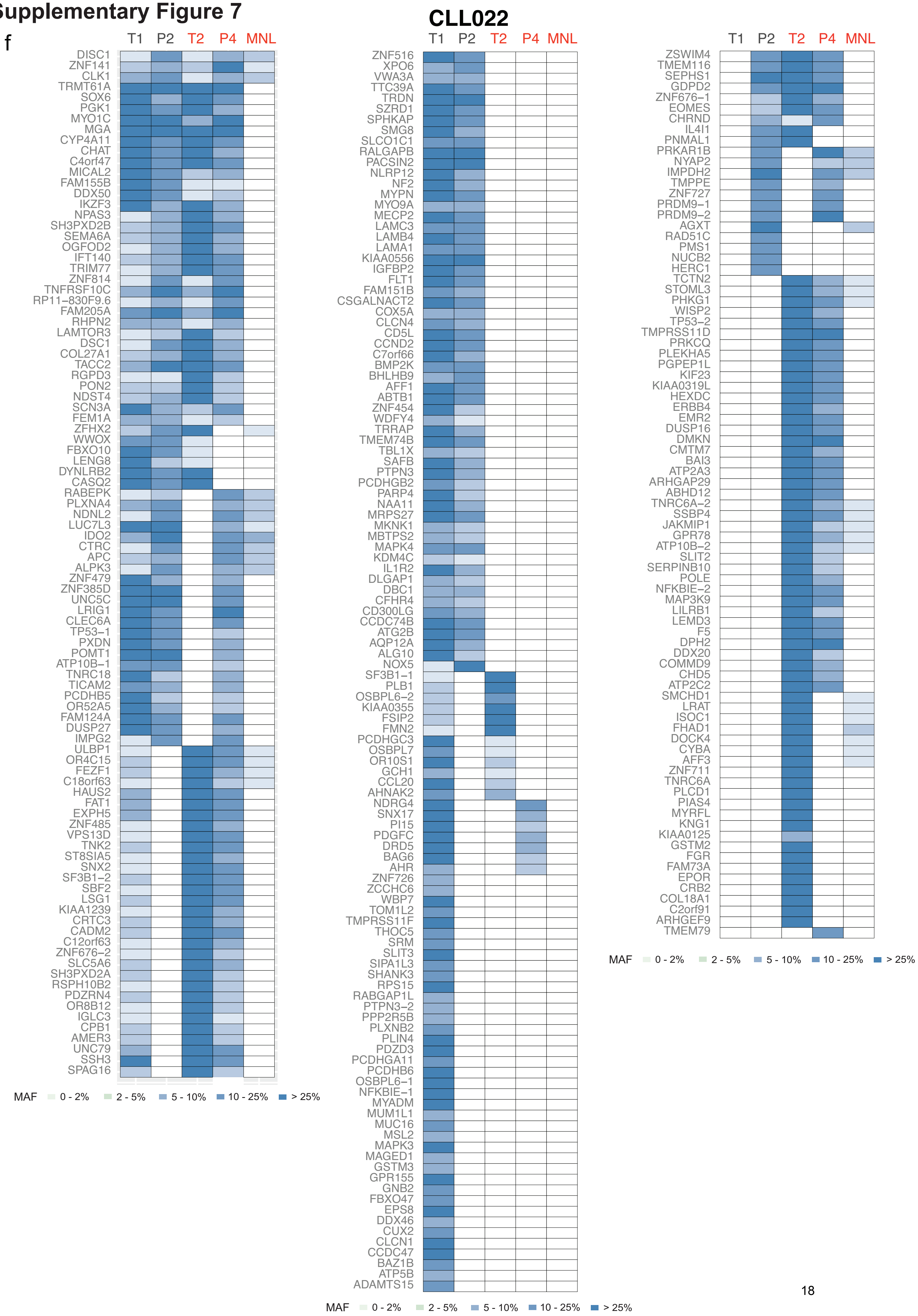

Supplementary Figure 7: Clinical assessment and ctDNA analysis in case CLL022

(a) A treatment timeline of case CLL022 highlighting the time-points of ctDNA assessment and bone marrow (BM) biopsies, respective to treatment with venetoclax. Whole exome sequencing (WES) was performed on DNA samples highlighted in red at baseline diagnosis (P1 and T1-BM) and at transformation to RS (P4 and T2-BM) in patient CLL022. Low-coverage whole genome sequencing (LC-WGS) was also performed on plasma (P1 and P4).

(b) ctDNA (left) and matched MNL DNA (right) dynamics were followed serially in case CLL022 and compared to clinical parameters of disease burden (PB lymphocyte counts and radiological measurement of lymphadenopathy). A clone bearing the mutation *TP53* R248Q was seen in ctDNA and matched MNL DNA, and displayed a gradual decrease over time in case CLL022, post the commencement of venetoclax. In contrast, two other mutations were observed to rise in ctDNA but not in MNL DNA (*TP53* C238S and *SF3B1* K666M) as the patient progressed to RS, 64 days before the clinical diagnosis of RS. The changes in ctDNA paralleled changes in the extent of lymphadenopathy on imaging, although the PB lymphocyte count was not elevated.

(c) B-allele frequency (BAF) plots showing copy number alterations (CNAs) in WES data from tissue samples at baseline (T1-BM) and at progression to RS (T2-BM) (left panel). Marked in yellow are the CNAs that were common between the two samples, green signifies CNAs that were unique to T1-BM and red signifies new CNAs that were unique to T2-BM at the time of RS diagnosis. Consistent with the copy number data, FISH analysis showed evidence of a 17p deletion (red probe) in T1-BM, not present in the larger transformed cells in T2-BM (right panel).

(d) FDG-PET imaging at RS diagnosis (Day 155) displayed extensive and intense marrow, liver and spleen metabolic abnormality combined with intense uptake in the upper abdominal and right paratracheal lymph nodes, highly suggestive of transformed disease.

(e) H&E staining of bone marrow biopsy (T1-BM) prior to the commencement of venetoclax displayed a high infiltration of CLL cells indicating heavy disease burden. Bone marrow biopsy performed on Day 155 post-treatment (time-point of RS diagnosis) was hypercellular with clear evidence of large cell transformation. High-resolution images (bottom panel) illustrate the clear difference in morphology of small, rounded CLL cells, in contrast to the

markedly disorganised morphology of the large transformed cells along with fibrovascular streaming. Scale bar – 100μm (top panel) and 20μm (bottom panel).

(f) A heat-map illustrating the distribution of predicted functional SNVs from WES at baseline (BM and P) and at progression to RS (BM, P and matched MNL shown in red) in CLL022.

Supplementary Figure 8

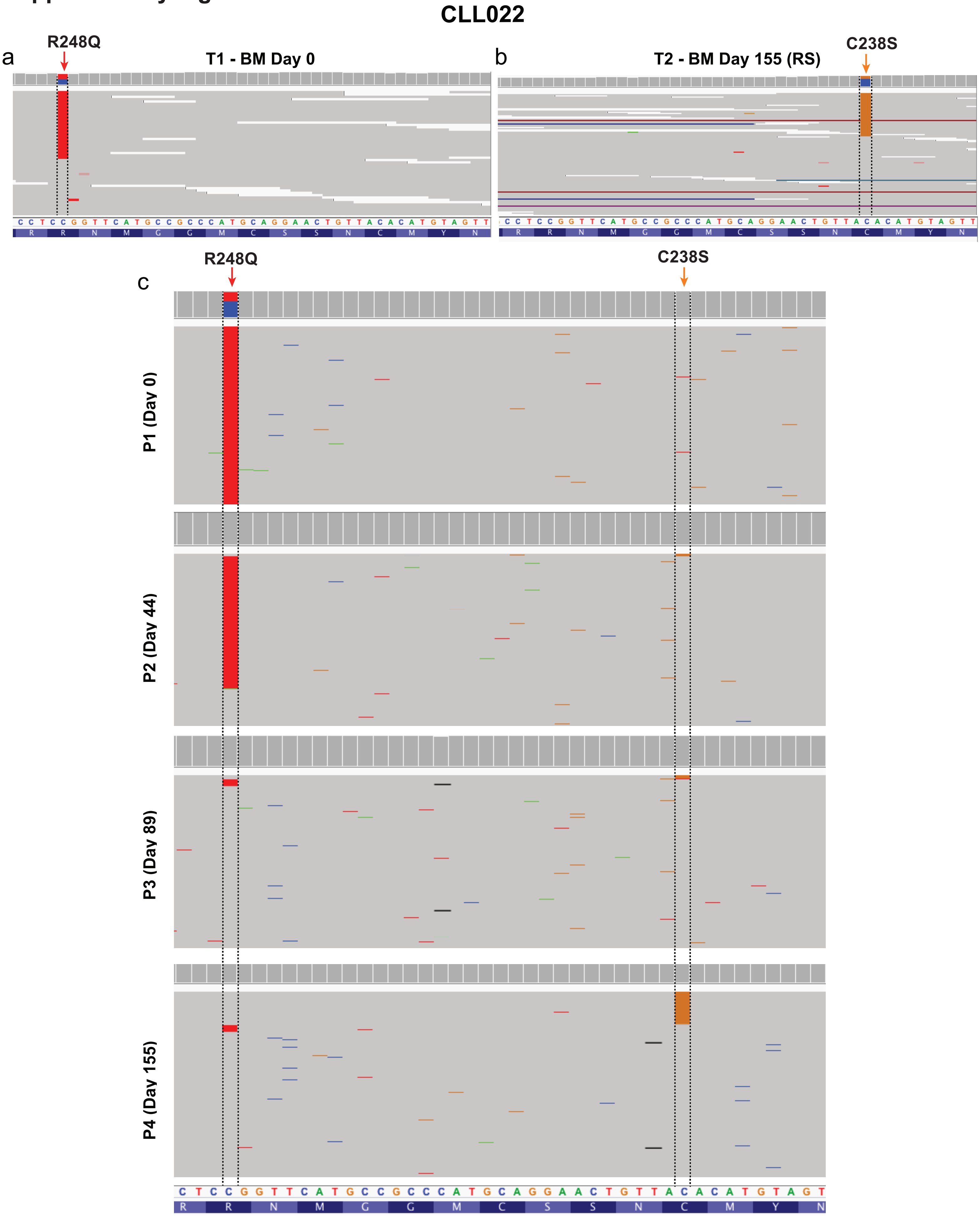

Supplementary Figure 8: Tissue and plasma analysis in CLL22 showing convergent evolution with emergence of a new *TP53* mutation at the time of RS

(a-b) IGV (Integrative genomics viewer) screenshots of WES data showing (a) the presence of a *TP53* R248Q mutation in the bone marrow (T1-BM) of CLL022 prior to the onset of venetoclax treatment and (b) the presence of a different *TP53* mutation, *TP53* C238S, from a separate sub-clone detected at the time of RS diagnosis (T2-BM).

(c) The dynamics of the two *TP53* mutations in serial plasma samples collected at baseline (Day 0), and subsequently at Days 44, 89 and 155 (RS diagnosis) post the onset of venetoclax treatment clearly demonstrate the decline of *TP53* R248Q over time, and the emergence of the *TP53* C238S mutation, detected as early as Day 44. The two mutations were on mutually exclusive sets of reads.

Supplementary Table 1: Summary of patient cohort prior to the onset of novel therapy

	Age (years)	Gender	Cytogenetics/ FISH at baseline	IGHV Status	Prior therapy	Novel therapy	RAI stage before novel agent	Response to novel therapy	Duration of novel therapy (months)	Gene/Mutation	Plasma MAF at baseline	PB-MNL MAF at baseline
**CLL004**	57	Male	17p-	Unknown	FCR, R-CHOP	Venetoclax	II	Richter's Syndrome	7	*TP53* (p.R280G)<br>*SF3B1*(p.H662D)<br>*ASXL1* 31022789 insT	0.10<br>0.10<br>0.02	0.17<br>0.11<br>0.02
**CLL006**	52	Male	13q-	Productive unmutated and unproductive	FCR, Chlorambucil, RCVP, ofatumamab.	Venetoclax	IV	PR	12	*MYD88* p.L265P	0.35	0.25
**CLL020**	63	Male	17p-, 13q-	Hypermutated	Rituxamab, chlorambucil + prednisolone	Venetoclax	IV	PR	12	*TP53* (p.R209fs)	0.29	0.64
**CLL022**	75	Female	17p-	Unmutated	FCR, Chlorambucil, Prednisolone, obinutuzumab	Venetoclax	IV	Richter's Syndrome	6	*TP53* R248Q<br>*TP53* C238S<br>*SF3B1* K666M	0.17<br>0.01<br>ND	0.70<br>0.01<br>0.01
**CLL029**	82	Female	17p-	Hypermutated	FCR, Chlorambucil, obinutuzumab	Venetoclax	II	PR	10	*TP53* C275Y	0.03	0.03
**CLL042**	85	Female	17p-, 13q-, 11q-	Unknown	Chlorambucil, rituximab	Venetoclax	III	CR	24	*TP53* D281G	0.76	0.91
**CLL050**	69	Male	17p-, 13q-	Unknown	FCR, CVP, Dexamethasone	Venetoclax	II	CR	17	*NOTCH1* P2514fs	0.39	ND
**CLL001**	69	Male	17p-, Trisomy 12	Unmutated	FCR, R-CHOP, R-VIC, ASCT, obinutuzumab	Ibrutinib	I	PR	19	*NOTCH1* (p.2514fs)<br>*TP53* (p.R337C)	0.04<br>0.04	0.02<br>ND
**CLL008**	71	Female	17p-, 13q-	Hypermutated	FCR, oblimersen, ofatumumab	Ibrutinib	II	PR	15	*TP53* (c.560-1G>A)	0.19	0.43
**CLL012**	73	Male	17p-, 13q-	unmutated	Alemtuzamab+ dexamethasone	Ibrutinib	I	PR	12	*TP53* p.R209Kfs	0.20	0.46
**CLL013**	70	Male	Abnormal 17q and complex	Unknown	FCR, ofatumamab	Ibrutinib	I	CR	24	*TP53* R248W	0.10	0.19
**CLL015**	84	Female	17p-, Trisomy 12	Unmutated	Chlorambucil, R-CEP. Ofatumamab	Ibrutinib	III	PR	10	*TP53* R234C	0.24	0.39
**CLL018**	58	Male	Trisomy 12, t(14:18)	Unmutated	FCR	Ibrutinib	IV	PR	12	*NOTCH1* (p.2514fs)	0.63	0.58
**CLL021**	68	Female	17p-, complex	Unmutated	FCR plus Lumiliximab, prednisolone	Ibrutinib	IV	PR	6	*TP53* G244S	0.01<br>(Day 28)	0.14<br>(Day 28)
**CLL040**	85	Male	17p-, hypodiploid	Unmutated	FCR	Ibrutinib	III	PR	36	*TP53* G245S	0.20<br>(Day 29)	0.83<br>(Day 504)
**CLL043**	54	Female	13q-	Unknown	FCR, allogenic transplantation	Ibrutinib	IV	PR	9	*SF3B1* R625C	0.28	0.46
**CLL053**	66	Male	17p-, complex	Unmutated	FCR	Ibrutinib	IV	PR	36	*TP53* V272M	0.14	0.35<br>(Day 28)
**CLL054**	65	Male	-13q	Unknown	FCR, Chlorambucil, RCVP	Ibrutinib	III	Richter's Syndrome	9	*TP53* C275F<br>*TP53* C275del	0.53<br>0.03	0.41<br>0.06
**CLL056**	51	Male	17p-	Unknown	Nil	Ibrutinib	III	PR	18	*TP53* E286K	0.86	0.96
**CLL058**	70	Male	17p-, 13q- ,complex	Unknown	FCR, chlorambucil	Ibrutinib	IV	PR	3	*TP53* I195T	0.88	0.86
**CLL064**	68	Male	13q-	Unknown	FCR, ofatumamab	Ibrutinib	IV	PR	7	*TP53* G245S	0.56	0.57
**CLL069**	67	Male	11q-,13q- ,complex	Unmutated	FCR	Ibrutinib	I	SD	5	*TP53* G244S	0.29	0.23
**CLL071**	67	Male	17p-, Trisomy 12	Unknown	FCR, RCHOP	Ibrutinib	I	PR	24	*NOTCH1* P2514fs	0.07	0.36
**CLL073**	75	Male	11q-, 13q-, complex	Unknown	FCR, RCHOP	Ibrutinib	III	PR	6	*SF3B1* Q903R	0.01	0.03
**CLL079**	67	Male	Normal	unknown	FCR, Chlorambucil	Ibrutinib	IV	PR	10	*TP53* R273C	0.07	0.06
**CLL078**	55	Female	17p-, 11q-	Unknown	None	Venetoclax	I	PR	13	None	ND	ND
**CLL068**	73	Male	11q-	Unknown	Chlorambucil, rituximab	Ibrutinib	II	PR	12	None	ND	ND
**CLL005**	47	Male	17p-	Unmutated	FCR, CHOP	Venetoclax	II	CR	27	None	ND	ND
**CLL036**	53	Male	17p-	Unmutated	FCR	Ibrutinib	II	CR	30	None	ND	ND
**CLL039**	73	Female	17p-, 11q-	Unknown	FCR, Dexamethasone, Obinutuzumab	Ibrutinib	IV	PR	12	None	ND	ND
**CLL033**	83	Female	17p-	Unknown	FCR, RCVP	Ibrutinib	IV	PR	28	None	ND	ND
**CLL049**	78	Male	del6q	Unknown	FCR, RCHOP	Venetoclax	IV	PR	35	None	ND	ND

Supplementary Table 2: Matched comparison between ctDNA, MNL DNA and disease assesment parameters

Patient	Day	PBL count (x10^9/L)	Radiology burden iwCLL (cm2)	% lymphocytes in Bone marrow aspirate	Bone Marrow B-lymphoid infiltrates	B2M mg/L	Plasma MAF	MNL MAF	Change in PBL (% of max)	Change in Radiology burden (% of max)	Change in Plasma MAF (% of max)	Change in MNL MAF (% of max)
CLL006	332	0.33	11.71				0.02	ND	0.73	16.46	4.50	0.00
CLL006	164	0.66	13.63	15	Yes		0.02	0.01	1.45	19.16	6.23	3.18
CLL006	80	0.37	15.17				0.02	0.01	0.81	21.32	6.47	2.89
CLL006	32	0.95	27.85				0.04	0.30	2.09	39.14	12.74	100.00
CLL006	-28	45.5	71.15	90	Yes	3.2	0.35	0.25	100.00	100.00	100.00	85.87
CLL001	362	2.7	6.65			2.7	ND	ND	100.00	44.04	0.00	0.00
CLL001	292	2.47	14.34	7	Yes	2.4	0.04	ND	91.48	94.97	100.00	0.00
CLL001	0	0.77	15.1	64	Yes	3.6	0.04	0.02	28.52	100.00	97.02	100.00
CLL013	177	6.14	11.53				0.02	0.65	100.00	34.50	9.77	100.00
CLL013	-14	1.85	33.42				0.23	0.19	30.13	100.00	100.00	29.77
CLL018	180	4.8	6.73				0.46	0.45	38.10	22.63	72.54	77.66
CLL018	0	12.6	29.74	86	Yes	4.3	0.63	0.58	100.00	100.00	100.00	100.00
CLL020	538	0.6	3.29				ND	ND	0.33	16.25	0.00	0.00
CLL020	333	0.8	5.1				0.03	ND	0.43	25.19	11.58	0.00
CLL020	169	35.4	12.66				0.09	0.37	19.21	62.52	32.37	57.24
CLL020	0	184.3	20.25	86	Yes	9.9	0.29	0.64	100.00	100.00	100.00	100.00
CLL029	125	1.77	4.76	16	Yes		0.01	ND	28.55	55.80	100.00	0.00
CLL029	291	6.2	5.03	27	Yes	4.3	ND	0.01	100.00	58.97	0.00	100.00
CLL029	524	2.81	8.53				0.01	ND	45.32	100.00	96.70	0.00
CLL029	606	1.51	3.45	13	Yes		ND	ND	24.35	40.45	0.00	0.00
CLL004	175	0.65	22.79	10	No		0.01	ND	32.99	22.42	6.60	0.00
CLL004	91	0.74	38.18				0.02	ND	37.56	37.56	20.01	0.00
CLL004	0	1.97	88.03	7	Yes		0.10	0.17	100.00	86.59	100.00	100.00
CLL004	233	1	101.66				0.08	ND	50.76	100.00	80.44	0.00
CLL042	253	1.57	1.98	7	Yes		0.02	0.01	3.17	8.15	1.96	1.24
CLL042	55	1.07	3.44				0.01	0.01	2.16	14.16	0.69	0.76
CLL042	5	49.57	24.29	97	Yes		0.76	0.91	100.00	100.00	100.00	100.00
CLL050	245	0.59	3.64	6	No		ND	ND	1.18	15.46	0.00	0.00
CLL050	77	0.88	6.37				ND	ND	1.75	27.05	0.00	0.00
CLL050	-34	50.2	23.55	75	Yes		0.51	ND	100.00	100.00	100.00	0.00
CLL053	336	3.18	2.04				0.08	0.11	19.63	28.02	33.21	32.63
CLL053	588	1.64	2.05				ND	ND	10.12	28.16	0.00	0.00
CLL053	756	1.4	2.11				0.01	0.02	8.64	28.98	4.68	6.69
CLL053	28	16.2	7.28				0.24	0.35	100.00	100.00	100.00	100.00
CLL054	318	11.6	10.25				0.01	0.21	42.18	8.39	6.36	46.87
CLL054	269	27.5	122.22	87	Yes		0.12	0.46	100.00	100.00	100.00	100.00
CLL008	182	44.3	2.91				0.12	0.35	100.00	34.32	74.28	80.23
CLL008	0	41.1	8.48				0.16	0.43	92.78	100.00	100.00	100.00

Maximal values in each case for each parameter denoted in red.

% of max = percentage of the maximal value at any time point in each case.

ND = Not Detectable

Supplementary Table 3: Comparison between ctDNA and MNL DNA across time points before and after novel therapy.

Before novel agent therpay

		**ctDNA**		
		Detected	Not detected	Total
**MNL DNA**	Detected	22	0	22
	Not detected	1	0	1
		23	0	23

After novel agent therapy

		**ctDNA**		
		Detected	Not detected	Total
**MNL DNA**	Detected	58	3	61
	Not detected	18	9	27
		76	12	88

Supplementary Table 4: List of Digital PCR Assays

Assay Name	Company	Gene	Chr	Genomic Location	Mutation	Protein change	Forward primer	Reverse primer	Probe 1 (VIC/HEX)	Probe 2 (FAM)	Size (bp)
Custom designed assays											
RPP30	Sigma	*RPP30*	10		-	-	AGATTTGGACCTGCGAGCG	GAGCGGCTGTCTCCACAAGT	TTCTGACCTGAAGGCTCTGCGCG	-	65
DRM	IDT	*MBD-like*	3R	5385569	-	-	ATGAACCCGAGCGTCACAAT	TGCTGCTGGCGTTGTTATTG	-	CCAAAGGGATGGCAGCGCGA	118
TP53_743	Sigma	*TP53*	17	7577538	c.743G>A	p.R248Q	TCCACTACAACTACATGTG	GAGTCTTCCAGTGTGATG	CGGCATGAACCGGAGGCCCATC	CGGCATGAACCAGAGGCCCATC	85
TP53_560	Invitrogen	*TP53*	17	757829	c.560-1G>A	-	CCCCAGGCCTCTGATTCC	TCCTTCCACTCGGATAAGATGCT	CTGATTGCTCTTAAGTCTGG	TGATTGCTCTTAGGTCTGG	73
NOTCH1_7541	Invitrogen	*NOTCH1*	9	139390649	c.7541_7542del	p.P2514fs*4	CTCGCCTGTGGACAACAC	CAGTCGGAGACGTTGGAAT	CCCGTCCCCTGAGT	TCCCGAGTCCCCTG	123
TP53_626	Invitrogen	*TP53*	17	7578222	c.626_627del	p.R209fs*6	CAGTTGCAAACCAGACCTCA	GTGGAAGGAAATTTGCGTGT	AGTGTTTCTGTCATCC	AAAAGTGTTTGTCATCC	100
PrimePCR ddPCR assays											
TP53_1009	BioRad	*TP53*	17	7573957	c.1009C>T	p.R337C	-	-	dHsaIS2505929	dHsaIS2505928	79
TP53_838	BioRad	*TP53*	17	7577039	c.838A>G	p.R280G	-	-	dHsaIS2505639	dHsaIS2505638	63
TP53_713	BioRad	*TP53*	17	7577507	c.713G>C	p.C238S	-	-	dHsaIS2503795	dHsaIS2503794	63
SF3B1_1873	BioRad	*SF3B1*	2	198267423	c.1873C>T	p.R625C	-	-	dHsaIS2505505	dHsaIS2505504	77
SF3B1_1984	BioRad	*SF3B1*	2	198267312	c.1984C>G	p.H662D	-	-	dHsaIS2501779	dHsaIS2501778	92
SF3B1_1997	BioRad	*SF3B1*	2	198267299	c.1997A>T	p.K666M	-	-	dHsaIS2502409	dHsaIS2502408	69
IGH ddPCR assays (Evagreen)											
CLL01-v1S38	IDT	IGH	14		-	-	GAGTCGAGTTACCATATCAGTAGAC	ACGTCCATACCGTAGTAGTAGTAC	-	-	162
CLL018-v1S41	IDT	IGH	14		-	-	AGTCGAGTCACCATATCAGTAGAC	CATACCGTAGTAGTAGTAGTAATTGC	-	-	144
CLL020-v1S42	IDT	IGH	14		-	-	CCGCGTCTGTGAAAGGCAGAT	GAGATCGAAGCTGTTCTTGCCTC	-	-	134
CLL043-v1S44	IDT	IGH	14		-	-	GCCGATTCACCATCTCCAGAG	GTCCATACCGGTCCTATCGCT	-	-	116

Supplementary Table 5: List of primers used for targeted sequencing

Assay Name	Chr	From	To	Forward	Reverse	Size
MYD88_AA_0001	3	38182554	38182668	GTTGAAGACTGGGCTTGTCC	TTCATTGCCTTGTACTTGATGG	114
MYD88_AA_0002	3	38182200	38182302	GCTGAACTAAGTTGCCACAGG	ACATTCCTTGCTCTGCAGGT	102
NOTCH1_AA_0001	9	139391655	139391853	CGTGCCTTGAGGTCCTTG	TGGACGAGTACAACCTGGTG	198
NOTCH1_AA_0002	9	139391271	139391464	TGACCATTCAAACTGGTGGA	GGCATCGGGCACCTGAAC	193
NOTCH1_AA_0003	9	139391098	139391290	AGCTCATCATCTGGGACAGG	TCCACCAGTTTGAATGGTCA	192
NOTCH1_AA_0004	9	139391377	139391574	GAGGTGGGCCAGTCTCAA	CCCATGGCTACCTGTCAGA	197
NOTCH1_AA_0005	9	139391548	139391734	AGGCCACGTCTGACAGGTAG	GTGCAGGGCAAGAAGGTC	186
NOTCH1_AA_0006	9	139390851	139391021	CCCAGTGGCTGCACGTCT	ACAGATGCAGCAGCAGAACC	170
NOTCH1_AA_0007	9	139390705	139390898	TGTCCACAGGCGAGGAGTA	GAGCTTCCTGAGTGGAGAGC	193
NOTCH1_AA_0008	9	139390524	139390723	TACTTGAAGGCCTCCGGAAT	TACTCCTCGCCTGTGGACAA	199
NOTCH1_AA_0009	9	139390594	139390698	ACCAGTCGGAGACGTTGG	CCAGCCACCAGCTACAGG	104
NOTCH1_AA_0010	9	139390455	139390619	ATCCACAGAGCGCACACA	CCCGCATTCCAACGTCTC	164
SF3B1_AA_0001	2	198267382	198267584	GCCAGGACTTCTTGCTTTTG	ACTCATGACTGTCCTTTCTTTGT	202
SF3B1_AA_0002	2	198267256	198267458	AGACAAAGTTACATTACAACTTACCA	TGTTGTAGCCTCTGCCCTG	202
SF3B1_AA_0003	2	198266773	198266956	CCATAAGGAGTTGCTGCTTCA	CTGCAGTTTGGCTGAATAGTTG	183
SF3B1_AA_0004	2	198266568	198266736	AAGAGGAATAAGATACCCAATAGCC	GAAGGGTATCCGCCAACAC	168
SF3B1_AA_0005	2	198266547	198266718	GTTGGCATATTCTGCATCCAT	CAGAGGAAAGGTAAATCCACCA	171
SF3B1_AA_0006	2	198266452	198266566	TCTGGAATAATTACCTTCAGCACA	TGGATGCAGAATATGCCAAC	114
SF3B1_AA_0007	2	198265363	198265505	AAATTTGCTTGACAACTAATATGCTTT	TGATCATAAACTTGAAGAACAACTGA	142
SF3B1_AA_0008	2	198266712	198266798	TCCTCTGTGTTGGCGGATA	TTGGCTGAAGCAGCAACTC	86
SF3B1_AA_0009	2	198266486	198266618	TTTCCTCATCAGGAGACTGGA	AAATAGGGTTTGGCTGCTTTC	132
SF3B1_AA_0010	2	198265440	198265588	TCTGTAGTCTGTTCTTGGAAAGCA	CTGAAAGATGAAGCCGAACAG	148
SF3B1_AA_0011	2	198265537	198265724	TCTCAATTGTCTCCATCACCA	TGATGTTGCTTTATTTCCTTGG	187
TP53_AA_0001	17	7579479	7579626	CAGCCTCTGGcATTCTGG	CCTGGTCCTCTGACTGCTCT	147
TP53_AA_0002	17	7579359	7579520	GGAAACCGTAGCTGCCCTG	AAGACCCAGGTCCAGATGAA	161
TP53_AA_0003	17	7579260	7579421	ATACGGCCAGGCATTGAAGT	CCTCCTGGCCCCTGTC	161
TP53_AA_0004	17	7577432	7577631	GGGGTCAGAGGCAAGCAG	CTTGGGCCTGTGTTATCTCC	199
TP53_AA_0005	17	7577003	7577187	TGTCCTGCTTGCTTACCTCG	GCCTCTTGCTTCTCTTTTCCT	184
TP53_AA_0006	17	7576908	7577075	CTGGTGTTGTTGGGCAGT	ATCTCCgCAAGAAAGGGGAG	167
TP53_AA_0007	17	7578425	7578594	TGTGCTGTGACTGCTTGTAG	TGCCCTGACTTTCAACTCTGT	169
TP53_AA_0008	17	7578361	7578525	AGCTGCTCACCATCGCTA	CCAACTGGCCAAGACCT	164
TP53_AA_0009	17	7578334	7578503	AACCAGCCCTGTCGTCTCT	GTGCAGCTGTGGGTTGATT	169
TP53_AA_0010	17	7578229	7578406	TCCAAATACTCCACACGCAAA	GCTGCCCCCACCATGAG	177
TP53_AA_0011	17	7578091	7578274	GAGAAAGCCCCCCTACTGC	AGCATCTTATCCGAGTGGAAGG	183
TP53_AA_0012	17	7576786	7576983	AGAAAACGGCATTTTGAGTGT	AAGGGTGCAGTTATGCCTCA	197
TP53_AA_0013	17	7576584	7576734	TCTGTATCAGGCAAAGTCATAGAA	GCCTCAAAGACAATGGCTCC	150
TP53_AA_0014	17	7579758	7579940	TTTCGCTTCCCACAGGTCTC	CAGCCAGACTGCCTTCCG	182
TP53_AA_0015	17	7573859	7574054	GGAATCCTATGGCTTTCCAACC	CCCCCTCCTCTGTTGCTG	195
TP53_AA_0016	17	7572850	7573030	GACCCAAAACCCAAAATGGC	TCCCTGCTTCTGTCTCCTAC	180
TP53_AA_0017	17	7579557	7579754	TCAAATCATCCATTGCTTGG	CCATGGGACTGACTTTCTGC	197
ATM__001	11	108117685	108117878	TTACAGGTGCTTATGAATCAACAAA	TGTGACATGACCTACTTACTGTACCTG	193
ATM_002	11	108121560	108121674	ACGATGCCTTACGGAAGTTG	ACCACGAAAGGTAATACACCAAA	114
ATM_003_R1	11	108180785	108180976	TTCTGTTAAGCAGTCACTACCATTG	TGAGCAGCACAAGACTGAGC	191
ATM_004	11	108180911	108181100	TGATGCTTTCTGGCTGGATT	CCTTATTGAGACAATGCCAACA	189
ATM_005	11	108186581	108186768	GGGAGCCAGATAGTTTGTATGG	CCACATTGCTTCGTGTTCAT	187
ATM_006	11	108186676	108186879	TGTTTATGAAGGAGTTATGTGTGTGTA	CACTAGTGATGGCTTTACCAAATC	203
BIRC3_001	11	102207588	102207741	TTTCACTGAAGAAGCAAACTGC	GATGACCACAAGGAATAAACACTATG	153
BIRC3_002	11	102201792	102201961	ATTAATGCTGCCGTGGAAAT	TCCTCAGTTGCTCTTTCTCTCTC	169
KRAS_001	12	25398124	25398305	ACTCATGAAAATGGTCAGAGAAACCT	AACTTGTGGTAGTTGGAGCTGGT	181
KRAS_002	12	25398209	25398391	TCTATTGTTGGATCATATTCGTCCAC	TGTATTAACCTTATGTGTGACATGTTCT	182
KRAS_003	12	25380205	25380372	TTATGGCAAATACACAAAGAAAGC	TCCAGACTGTGTTTCTCCCTTC	167
ASXL1-Exon12-06	20	31022716	31022902	GGGCTACAGTTGGACTCACA	CAGGAAGTGGTGCCAGACTC	186

Supplementary Table 6: Dominant IGHV sequences for CLL001, CLL018, CLL020 and CLL043

Patient	Dominant IgH sequence	Length	Vfamily	Dfamily	Jfamily	Homology to germline	Status
CLL001	TGATTACTACTGGAGTTGGATCCGCCAGCC<br>CCCAGGGAAGGGCCTGGAGTGGATTGGGT<br>ACATCTATTACAGTGGGAGCACCTACTACAA<br>CCCGTCCCTCAAGAGTCGAGTTACCATATCA<br>GTAGACACGTCCAAGAACCAGTTCTCCCTG<br>AAGCTGAGCTCTGTGACTGCCGCAGACACG<br>GCCGTGTATTACTGTGCCACCAACTCTCGG<br>GGGGTATTACTATGGTTCGGGGAGTTGGGG<br>TACTACTACTACGGTATGGACGTCTGGGGCC	312	Homsap IGHV4-30-4*01 F, or Homsap IGHV4-30-4*02 F	Homsap IGHD3-10*01 F	Homsap IGHJ6*02 F	100.00%	Unmutated
CLL018	TTACTACTGGGGCTGGATCCGGCAGCCCCC<br>AGGGAAGGGGCTGGAGTGGATTGGGAGTA<br>TCTATCATAGTGGGAGCACCTACTACAACCC<br>GTCCCTCAAGAGTCGAGTCACCATATCAGTA<br>GACACGTCCAAGAACCAGTTCTCCCTGAAG<br>CTGAGCTCTGTGACCGCCGCAGACACGGC<br>CGTGTATTACTGTGCGAGGATCAATTACTAT<br>GGTTCAAGCAATTACTACTACTACTACGGTAT<br>GGACGTCTGGGGCCAAGGGACCAC	297	Homsap IGHV4-38-2*01 F	Homsap IGHD3-10*01 F	Homsap IGHJ6*02 F	100.00%	Unmutated
CLL020	CTACATGGACTGGGTCCGCCAGGCTCCAG<br>GGAAGGGGCTGGAGTGGGTCGGCCGTATT<br>AGAAACAAGCCTAAAAGTTACACCACAGTGT<br>ACGCCGCGTCTGTGAAAGGCAGATTCACCA<br>TCTCAAGAGATGATTCACAGAGCTCACTGTA<br>TCTGCAAATGGACAGCCTGACAAGCGAGGA<br>CACGGCCGTCTATTACTGTGTTAGAGGCAA<br>GAACAGCTTCGATCTCTGGGGCCGTGGCG<br>CCCT	273	Homsap IGHV3-72*01 F	Homsap IGHD5-24*01 ORF	Homsap IGHJ2*01 F	91.30%	Mutated
CLL043	TGCAGCCTCTGGACTCACCTTCAGTAGTTAT<br>GACATGAACTGGGTCCGCCAGGCTCCAGG<br>GAAGGGGCTGGAGTGGGTGTCATCCATTAG<br>TAGTAGTAGTTACATATACTACGCAGACTCAG<br>TAAAGGGCCGATTCACCATCTCCAGAGACA<br>ACGCCAAGAACTCACTGTATCTGCAAATGAA<br>CAGCCTGAGAGCCGAGGACACGGCTGTGT<br>ATTACTGTGCGAGCGATAGGACCGGTATGG<br>ACGTCTGGGGCCAAGGGACCAC	264	Homsap IGHV3-21*01 F, or Homsap IGHV3-21*02 F	Homsap IGHD2-2*01 F	Homsap IGHJ6*02 F	97.96%	Unmutated (borderline)
